# Instrumental Activities of Daily Living (iADL) Limitations in Europe: An Assessment of SHARE Data

**DOI:** 10.3390/ijerph17207387

**Published:** 2020-10-10

**Authors:** Diana Portela, Marta Almada, Luís Midão, Elísio Costa

**Affiliations:** 1 Agrupamento de Centros de Saúde (ACES) Entre Douro e Vouga I—Feira Arouca, Faculdade de Medicina da Universidade do Porto, 4200-450 Porto, Portugal; di.portelasilva@gmail.com; 2 Applied Molecular Biosciences Unit (UCIBIO) Requimte (Rede de Química e Tecnologia), Porto4Ageing, Faculdade de Farmácia da Universidade do Porto, 4050-047 Porto, Portugal; martassalmada@gmail.com (M.A.); luismidao@gmail.com (L.M.); 3 Laboratory of Biochemistry, Department of Biological Sciences, University of Porto, 4050-313 Porto, Portugal

**Keywords:** iADLs, SHARE, ageing, public health

## Abstract

This study aims to evaluate the instrumental activities of daily living (iADLs) limitations in Europe and its association with socio-demographic characteristics, economic parameters and physical and mental health status. We used data from the wave 6 of SHARE database. Individuals were classified as having either none or one or more limitations on iADLs. Participants aged 65 or more years who answered all questions for the variables included in this work were selected. A total of 54.8% of participants were female and had a mean age of 74.37 (SD = 7.08) years. A global prevalence of 1 or more iADLs in Europe was shown to be 23.8% and more prevalent in women than in men (27.1% vs. 17.6%) and in people aged 85 years or more (51.5%). Older age, female gender, lower education, physical inactivity, frailty, having two or more chronic diseases, presence of depression, polypharmacy, poor self-perception of health and lower network satisfaction were found to be factors associated with the presence of 1 or more iADLs limitation. This study highlights the burden of iADLs limitations at the European level. These are based on a multidimensional biopsychosocial model and are associated with both health conditions and environmental factors. This intersection between the physical and social world underscores its potential as a health indicator and can, to some extent, explain some of the pronounced differences seen among European countries. Different inter-tasks can also stress different dimensions of health indicators in distinct and specific groups of individuals. Minimizing the impact of iADL limitations can improve the quality and sustainability of public health systems.

## 1. Introduction

In recent decades, a demographic transition in Europe has been reflected by increased longevity and decreased birth rates, followed by the expansion of comorbidities and physical disability (PD) in older people [1,2]. The growing requirement for formal and informal care, as a consequence of PD in late adulthood, has increased the demand for healthcare services. This has led to financial expenditure increases associated with the diminished labour force and increased pensions, public costs and health and long-term care [3,4,5].

The Worldwide Classification of Functioning, Disability and Health (ICF) created an agreement to group the incapacity and dependence of older populations and access and quantify health system performance [6,7]. ICF is a multidimensional health tool that provides a framework and classification system that facilitates the measurement of the needs, performance and effectiveness of health care systems in distinct areas. This is particularly relevant with respect to disability, where the diagnosis alone does not predict functional outcomes. This is because outcomes are a result of interactions between the physical and social world [8]. Thus, the ICF provides reliable data for policy development, economic analysis, research use and interventional studies. One major innovation of this tool has been the use of environmental factors that encompass the physical, social and attitudinal environment in which people live and conduct their lives, highlighting the importance of the scientific understanding of disability [7].

Activities of daily living (ADLs) measurements estimate essential faculties needed for survival while instrumental activities of daily living (iADL) are described as important competencies required for living independently in a community [9]. These concepts are addressed through a questionnaire that includes six domains of ADLs, which are assessed using Katz Index values, and eight domains of iADLs, which are described using the Lawton–Brody Scale [10,11]. ADLs and iADLs measuring PD ranges in a biopsychosocial interface clearly impact socio-economic and care resources. Functional decline may be prevented or delayed by paying increased attention to healthy longevity indices. The monitoring of PD promotes creation of efforts and policies that aim to diminish mortality, institutionalisation and maintain the good health and high functioning of individuals into old age [12]. 

Old age, the female gender, physical inactivity, chronic diseases, high levels of drug consumption, the prevalence of pain, high or low body mass index (BMI), depression, poor self-perception of health, smoking and alcohol consumption and lower social participation have been described as factors associated with an increased incidence of disability in older people. These parameters are believed to be vitally important for active ageing. Additionally, previous studies have shown that disability in older people is influenced by living environment architectural, communication, social and other barriers and obstacles, such as ramps, handrails, elevators or appropriated signage, that promote healthy living and may actively influence the ageing process [8,13,14,15,16].

A key effect of iADLs on self-management and self-care activities has been that iADLs limits are expected to precede ADLs shortfalls. This outlines a need for interventions capable of limiting the onset of ADLs dependence [1,3,6]. Indeed, this study aims to evaluate iADLs limitations in 17 European countries and Israel, and their associations with sociodemographic characteristics, economic parameters, physical status, psychological domain and lifestyle using the Survey of Health, Ageing, and Retirement in Europe (SHARE), a population-based survey.

## 2. Materials and Methods

This work used data from the SHARE Wave 6 database. The SHARE project is a European cross-national panel database, which includes detailed questions regarding demographics, health, social and economic status from representative samples of the community-based population. The Wave 6 version of SHARE includes data collected from 17 European countries and Israel. Designed based on the US Health and Retirement Study and the English Longitudinal Study of Ageing, SHARE contains data from a very large population, and has a harmonised cross-national design, which provides a dynamic picture of ageing in Europe. A detailed international comparison, with a large number of dimensions and a description of SHARE data and methodology has been published and is available to registered users on the SHARE website (http://www.share-project.org). SHARE is a cross-sectional analysis study and Wave 6 contains data collected in 2015 from 68,231 community-dwelling individuals between the age of 24 and 103.

### 2.1. Dependent Variable—Limitations of iADLs

Functional limitations of iADLs served as dependent variables. The iADLs index described the number of an individual’s limitations to instrumental activities of everyday life by accessing eight domains [11]. Participants were asked whether they had any difficulty doing each of the following everyday activities: “doing work around the house or garden”, “leaving the house independently/accessing transportation”, “shopping for groceries”, “doing personal laundry”, “managing money”, “preparing a hot meal”, “taking medications” and “making telephone calls”. Individuals were instructed to exclude any difficulties expected to last less than three months. This variable was dichotomised as follows: ‘no iADL limitation’ and ‘one or more iADLs limitations’.

### 2.2. Explanatory Variables

As a result of the great volume of information within the SHARE database, we were able to include a diverse set of putative explanatory variables in our study. Among these were variables capable of reflecting the biopsychological environment associated with disability.

Socio-demographic variables that were included in the study were age, gender and years of education. Age was categorised into three classes (65–74, 75–84 and ≥85). The gender response generated a dichotomous variable, male or female. The variable “number of years of education” was used as a continuous variable ranging from 0 to 25 years.

In parallel with education, the variable, “shortage of money,” was used to address financial and socio-economic status. “Shortage of money” was accessed on the basis of the following question: “How often do you think that shortage of money stops you from doing the things you want to do?” Four possible responses to the question were offered, as follows: “Often”, “Sometimes”, “Rarely” and “Never”.

To assess the physical status of participants, variables such as physical inactivity, number of chronic diseases, polypharmacy, body mass index (BMI) and frailty status were included. Physical inactivity was assessed as follows: “How often do you engage in activities that require a moderate level of energy such as gardening, cleaning the car, or doing a walk?” and “We would like to know about the type and amount of physical activity you do in your daily life. How often do you engage in vigorous physical activity, such as sports, heavy housework, or a job that involves physical labour?” Options for answering both questions were as follows: “More than once a week”, “Once a week”, “One to three times a month” and “Hardly ever, or never”. Physical inactivity was defined as never or almost never engaging in moderate or vigorous physical activity and respondents that selected either “One to three times a month” or “Hardly ever, or never” as answers to the questions were considered physically inactive. The variable “number of chronic diseases” was based on the number of chronic diseases reported by each individual, which was dichotomised by less than or equal to two or more than two chronic diseases. Polypharmacy was assessed with the following question: “do you take at least five different drugs on a typical day?” Participants were asked to include drugs prescribed by the doctor, drugs bought without prescription and dietary supplements such as vitamins and minerals. Individuals had the option of selecting a dichotomous “yes” or “no” response. The continuous variable BMI was transformed into a discrete variable by grouping the BMI into four discrete ranges (below 18.5, 18.5–24.9, 25–29.9 and more than 30 kg/m^2^). Frailty was accessed as previously described [17]. For describing psychological domain and lifestyle, we included four variables in the analysis, as follows: “self-perceived health”, “life has meaning”, “network satisfaction” and “depression”. Self-perceived health was assessed by asking respondents to complete the sentence “Would you say your health is...” with one of the following options: “excellent”, “very good”, “good”, “fair” and “poor”. The variable “life has meaning” was derived from the question “How often do you feel that your life has meaning?” with four possible responses: “often”, “sometimes”, “rarely” or “never”. The variable “Network satisfaction” was assessed via the following question: “Overall, how satisfied are you with the [relationship that you have with the person/relationships that you have with the persons] we have just talked about? Please answer on a scale from 0 to 10, where 0 means completely dissatisfied and 10 means completely satisfied” [18]. Depression was assessed based on the EURO-D scale. The EURO-D symptom scale measures current levels of depression based on twelve items: depressed mood, pessimism, suicidality, guilt, sleep, interest, irritability, appetite, fatigue, concentration, enjoyment and tearfulness. For the purpose of our study, we used a continuous variable range that spanned from 0 “not depressed” to 12 “very depressed”.

### 2.3. Statistical Analysis

To address the prevalence of iADLs limitations within the European population assessed, we first performed a descriptive analysis. Given the multilevel structure of data, with individuals nested in each country, we used a multilevel logistic regression and considered iADLs limitations as the dependent variable. In the first phase, we used a multilevel univariable logistic regression model with dependent variables and assessed each covariate to identify potential factors associated with iADLs limitations (unadjusted model). Only significant covariates (*p* < 0.05) were included in a final multilevel multivariable logistic regression model (adjusted model). Country was considered a random effect in the analysis. The final model was composed only of significant covariates, that were selected using a backward selection method. Odds ratios and 95% confidence intervals (CI) were reported. The significance level was set at 0.05. All analyses were performed using IBM SPSS (version 25, IBM, Armonk, NY, USA) software.

## 3. Results

Of a total of 68,231 individuals that participated in wave 6 of the SHARE survey, we selected those aged 65 years or older who answered all questions for the variables included for analysis, yielding a total of 27,491 individuals. Participants had a mean age of 74.37 (SD = 7.08) years and 54.8% were female. The overall prevalence of individuals who reported difficulties performing at least one iADL was 23.8%.

Table 1 shows the standardised prevalence of possessing one or more iADLs limitation according to country, gender and age group.

The presence of at least one iADL limitation was more prevalent in women than men (27.1% vs. 17.6%, respectively) for all age groups and in every country studied, except for people living in Switzerland between 65–74 years (9.9% (9.3–10.6) and 9.1% (8.6–9.7%) for males and females, respectively). The presence of at least one iADL limitation was most prevalent among the oldest age group (85 or more years). The highest prevalence of iADLs limitations in all age groups was observed in Portugal (29.9 (29.3–30.4%)); however when stratifying by age group, Poland had the highest prevalence of iADLs within individuals between 75–84 years (42.2(41.1–43.3%)) and Israel had the highest prevalence of iADLs within individuals aged 85 or more years old (68.6(66.3–70.9%)).

To determine which groups of individuals experienced the most iADLs limitations, we summarised iADLs distribution by country (Table 2). Among the eight tasks included in this analysis, “Doing work around the house or garden” appeared with greatest prevalence throughout all countries (14.6%). A comparison of its prevalence between individual countries revealed that it was most prevalent in Poland (22.9%) and least prevalent Italy (7.6%). “Taking medications” and “Telephone calls” were the most seldomly experienced iADLs.

Using a univariate model (Table 3), we found a significant association between one or more iADLs limitation and all studied explanatory variables.

The multivariate analysis (Table 3) revealed that an observed reduced prevalence of iADLs limitations was associated with the male gender (OR = 0.680 (0.633–0.730)). Moreover, per each year of education, a decrease was found in the prevalence of iADL limitation (OR = 0.976 (0.967–0.985)).

Further, the prevalence of iADLs limitations was determined to be reduced in individuals of younger age groups 65–74 years (OR = 0.256 (0.229–0.286)) and 74–84 years (OR = 0.456 (0.409–0.508)) compared to individuals greater than or equal to 85 years old. Similarly, individuals that performed physical activity (OR = 0.404 (0.365–0.446)); participants presenting less than two chronic diseases (OR = 0.676 (0.621–0.736)); those who reported excellent (OR = 0.176 (0.127–0.246)), very good (OR = 0.210 (0.176–0.252)), good (OR = 0.304 (0.271–0.342)) or fair (OR = 0.507 (0.459–0.560)) self-perceived health status when compared with those presented poor self-perceived health status; and those that reported robust (OR = 0.237 (0.206–0.273)) and pre-frail (OR = 0.423 (0.381–0.470)) status when compared with individuals revealing a frail status tended to possess fewer iADLs limitations. Compared with obese individuals, individuals with weight classified as normal (OR = 0.889 (0.810–0.976)) and overweight (OR = 0.863 (0.793–0.940)) had a lower prevalence of iADLs limitations. However, a higher prevalence of iADLs limitations was observed in underweight (OR = 1.335 (0.985–1.809)) than obese individuals. The prevalence of iADLs was greater in individuals with polypharmacy (OR = 1.608 (1.493–1.731)), with depressive symptomatology (OR = 1.082 (1.063–1.101)) and in individuals who reported often (OR = 1.153 (1.039–1.281)) or sometimes (OR = 1.101 (1.000–1.105)) shortage of money when compared with individuals who never revealed a shortage of money. Moreover, the explanatory variables including “network satisfaction” and “life has meaning” did not significantly affect prevalence using the adjusted model.

## 4. Discussion

European countries currently face an adverse demographic trend that has occurred in parallel with the increased longevity of the population; leading to an increased prevalence of iADLs limitations. This has negative consequences, especially with regards to the sustainability of pension and health systems [19,20]. This pattern is accompanied by a growing number of people that endure confinement, perform self-care and self-management, experience iADL limitations, and show an increased reliance on external care. Currently, it is a public health issue to recognise lifestyle risk factors and to understand and create financial sustainability by addressing possible targets for the prevention of experiencing lifestyle limitations. Thus, in order to fill a literature gap, we performed an observational study that assessed the prevalence of iADLs limitations in European countries and Israel, and factors associated with iADLs limitations.

We found that the prevalence of iADLs limitations in older populations varied across countries with gender and age. As shown in Table 2, Israel was unique; these findings were likely caused by cultural and immigration demographic shifts, rather than biological differences between individuals living in Israel and Europe. It is expected that different health- and quality-of-life-related risk factors across Europe would impact disability among older adults. Moreover, the prevalence of chronic diseases, healthcare systems architecture, socioeconomic inequalities, different educational levels, development of social policies, environmental factors and population structure and vulnerability have the potential to influence perception of individual’s iADLs limitations [7,21], which can, to some extent, explain some of the pronounced differences seen among European countries. Specific analyses of inter-task differences revealed that housekeeping is a commonly observed iADL limitation, suggesting that the capacity to perform tasks related to housekeeping is among the first iADLs to deteriorate. Different inter-task can stress different dimensions of health indicators in distinct and specific groups of individuals. Furthermore, using the telephone and taking medications appeared to exhibit some resistance to impairment, and it was suggested that they reflect increased vulnerability to cognitive function decline [22].

Our results confirmed previous findings that older age, being female, and having less education are associated with a higher prevalence of iADLs limitations. To this end, it is not surprising that ageing, as a natural process, will be associated with disability, and therefore impact iADLs limitations’ presence and perception [16,23].

Life expectancy in Europe is higher in women, which makes them more susceptible to functional decline in late life. Social participation also encourages individuals to practice iADLs [24]. Therefore, significant differences observed in labour market participation have the potential to affect women disproportionately [24,25]. Moreover, the propensity of women with decreased muscle mass and experiencing early chronic onset events may explain observed increases in levels of PD and the increased numbers of iADLs limitations reported [16,26,27]. iADLs prevalence also affected the nature of the activities studied, which, in the older population selected, tend to be practiced most frequently by women [12].

The relationship between low socio-economic status, education and health status throughout the course of one’s life has been well established [28,29,30]. Financial status affects health in a number of ways, including through the health literacy of preventative medicine or through health care accessibility [31]. In fact, economic disadvantages, health inequalities and risky behaviours during an individual’s life course are reflected in increased risk of disability in old age. This occurs due to poor health choices, risky behaviours and the lack of health-related treatment opportunities [3,28,32,33]. Hence, fewer years of education and frequent money shortage were associated with an increased prevalence of iADLs limitations. Socio-economic and environmental factors can be tackled by policy enforcement, the development of regulations and technology or interventions to minimise the perception of disability using social and health resources. This type of access may also partially explain some of the differences observed at the social or institutional level, by highlighting its socioeconomic inequality association [34]. Thus, evidence regarding disability burden may indicate that society can effectively prevent and delay activity limitations to promote the inclusion and participation of disadvantaged individuals.

Increased longevity leads to the enhanced occurrence of chronic diseases, comorbidities and geriatric syndromes [35]. As expected, physical inactivity, multiple comorbidities, polypharmacy, obesity, being underweight and frailty were all associated with the presence of more than one iADLs limitation, which reflects the multi sectorial geriatric dimension of PD [17,22,36,37]. Though a wide range of cardiovascular, oncologic or orthopaedic pathologies can disrupt functional decline and iADLs performance, they also have a cumulative effect on health and are linked to polypharmacy and its associated side effects, physical activity restrictions, poor nutritional status, muscle mass loss and frailty [38,39]. Obesity causes movement difficulties with the capacity to cause health problems as a result of additional strain prior to functional limitation [40]. Being underweight, which leads to frailty syndrome and sarcopenia development, produced a similar outcome [41].

The accumulation of disabilities is not restricted to physical criteria or related indicators. Cognitive deterioration strongly impacts iADLs performance [22,42]. Exceptional consideration should be provided regarding the impact of cognitive ability. Activity restriction promotes depression in people. Simultaneously, cognitive decline contributes to already-prevailing functional limitations [43,44,45]. Functional capacities are assessed in the diagnosis of dementia using multiple diagnostic criteria [46]. Additionally, consistent findings regarding the type and frequency of social participation and its association with iADLs limitations have been reported [24]. Many mechanisms explain this relationship, since social participation encourages individuals to remain active, promotes health literacy through access to relevant information and has positive psychological effects [23,24]. The results of our study show that subjective perceptions such as self-perceived health, life satisfaction and depression have an important relationship with iADLs limitations. Nevertheless, “network satisfaction” and “life has meaning” were excluded from the model, since their effects were deemed insignificant after adjusting for all variables.

The SHARE database provides important information since it includes a large, cross-national, European cohort of individuals aged more than 50 years. Data were collected in accordance with standardised protocols and have been harmonised with other ageing studies, which permitted the generalisation of findings. Some limitations of the study should be cited. First, the impact of uncontrolled variables that might influence the presence of iADLs limitations or act as confounders was not evaluated. Second, cross-country comparisons of the prevalence of iADLs limitations are known to be limited by contextual, social and cultural differences between countries. This was controlled by adjusting data in accordance with country of residence [47]. Additionally, the sampling procedure may have influenced the results. The sample size of the study was significantly reduced due to the limited number of SHARE participants who answered all the of questions included in the study. It is also important to consider bias regarding the selective exclusion of unhealthier populations from our study. This occurred since they are less able to collaborate and may have altered findings regarding the impact of iADLs limitations. Finally, studies on self-reported measures may be contradictory. However, in the absence of an objective performance measure for iADLs limitations, it may be beneficial to avoid conclusions but raise hypotheses by examining approaches used by all researchers [48].

In conclusion, since successful performance in ADLs and iADLs are important health indicators, the present study highlights the burden of iADLs limitations in Europe. It also emphasises that disability is not unidirectional. Instead, it is a multidimensional concept, and its aspects are correspondingly combined in the definition of health determined by World Health Organisation [41]. Therefore, information regarding the disability burden of various diseases and health conditions is needed. It is also critical to address modifiable health determinants by improving health literacy and reducing health inequalities between different economic groups [49]. To this end, the financial effect of functional limitations and cost-effectiveness of resources when compared to the expense of adjusting standard services and social environments should be examined to guarantee that society is able to prioritise and effectively prevent iADLs limitation progression. Routine inclusion of iADLs limitations as a variable in public health studies can improve surveillance and be crucial for programme planning, policy and decision making, and the implementation and assessment of these interventions.

## 5. Conclusions

iADLs limitations described in this work were based on a multidimensional biopsychosocial model and were associated with both health conditions and environmental factors. This intersection between the physical and social world underscores our need for information regarding disability and iADL limitations. This is because minimising the impact of iADLs limitations can improve the quality and sustainability of public health systems by improving efficiency on development and implementation of strategies that modify and re-build social environments.

## Figures and Tables

**Table 1:** Prevalence of one or more iADLs limitation according to country, gender, and age group. iADLs (Instrumental activities of daily living); CI (confidence interval).

Country	Prevalence of 1+ iADLs Limitation (%)											
	Overall				Male				Female			
	Standardised Prevalence Rates (95% CI)				Standardised Prevalence Rates (95% CI)				Standardised Prevalence Rates (95% CI)			
	Overall	65–74	75–84	≥85	Overall	65–74	75–84	≥85	Overall	65–74	75–84	≥85
Austria	26.7 (26.2–27.2)	14.7 (14.2–15.2)	32.8 (31.9–33.8)	61.1 (58.9–63.3)	21.9 (21.2–22.5)	11.2 (10.6–11.9)	27.3 (26.1–28.6)	52.4 (49.6–55.3)	30.1 (29.4–30.9)	17.4 (16.6–18.3)	36.7 (35.3–38.2)	66.3 (63.2–69.6)
Germany	21.2 (20.8–21.7)	12.5 (12.0–13.0)	23.7 (22.9–24.6)	51.4 (49.5–53.5)	19.4 (18.8–20.0)	10.9 (10.2–11.5)	20.8 (19.7–21.9)	51.5 (48.7–54.4)	23.2 (22.6–23.9)	14.1 (13.4–14.9)	27.2 (25.9–28.5)	51.4 (48.6–54.3)
Sweden	17.3 (16.9–17.7)	10.5 (10.1–10.9)	20.5 (19.7–21.3)	37.5 (35.8–39.2)	14.2 (13.7–14.8)	7.8 (7.3–8.4)	17.4 (16.4–18.4)	33.0 (30.8–35.3)	20.1 (19.5–20.8)	12.8 (12.1–13.5)	23.6 (22.4–24.8)	41.9 (39.4–44.5)
Spain	19.7 (19.3–20.1)	10.3 (9.8–10.7)	23.8 (23.0–24.6)	48.7 (46.8–50.7)	14.4 (13.9–15.0)	6.7 (6.2–7.2)	18.0 (17.0–19.1)	37.6 (35.2–40.1)	24.6 (23.9–25.3)	13.5 (12.8–14.2)	29.4 (28.1–30.8)	58.5 (55.5–61.5)
Italy	17.2 (16.8–17.6)	8.5 (8.1–8.9)	21.6 (20.8–22.4)	42.5 (40.7–44.3)	10.6 (10.1–11.0)	3.3 (3.0–3.7)	16.0 (15.1–17.0)	26.7 (24.7–28.8)	23.8 (23.2–24.5)	13.0 (12.3–13.7)	27.4 (26.1–28.7)	60.4 (57.4–63.5)
France	23.3 (22.9–23.8)	12.8 (12.4–13.3)	27.7 (26.8–28.6)	56.0 (53.9–58.1)	16.4 (15.9–17.0)	8.6 (8.1–9.2)	21.3 (20.2–22.4)	36.7 (34.4–39.2)	28.0 (27.2–28.7)	16.2 (15.5–17.0)	32.4 (31.0–33.8)	65.8 (62.7–69.1)
Denmark	20.2 (19.8–20.7)	12.4 (11.9–12.9)	23.8 (23.0–24.6)	43.9 (42.1–45.8)	17.2 (16.6–17.8)	9.9 (9.3–10.5)	18.5 (17.5–19.6)	44.2 (41.7–46.9)	23.0 (22.4–23.7)	14.9 (14.2–15.6)	28.3 (27.0–29.6)	43.8 (41.2–46.4)
Greece	29.7 (29.1–30.2)	18.5 (17.9–19.1)	37.8 (36.7–38.8)	55.6 (53.6–57.7)	23.7 (23.0–24.4)	15.2 (14.5–16.0)	25.1 (23.9–26.3)	55.6 (52.7–58.6)	34.8 (34.0–35.7)	21.4 (20.6–22.3)	48.5 (46.8–50.2)	55.7 (52.8–58.7)
Switzerland	16.5 (16.1–16.9)	9.5 (9.1–10.0)	17.7 (17.0–18.4)	42.6 (40.8–44.4)	14.2 (13.7–14.7)	9.9 (9.3–10.6)	11.8 (11.0–12.7)	38.2 (35.8–40.7)	18.5 (17.9–19.1)	9.1 (8.6–9.7)	23.3 (22.1–24.5)	45.2 (42.6–47.9)
Belgium	28.9 (28.4–29.5)	18.9 (18.3–19.5)	33.2 (32.2–34.2)	60.1 (58.0–62.3)	23.9 (23.2–24.5)	15.7 (15.0–16.5)	26.5 (25.3–27.8)	51.0 (48.2–53.8)	33.2 (32.4–34.0)	21.6 (20.8–22.5)	38.6 (37.1–40.1)	68.1 (64.9–71.4)
Israel	29.1 (28.6–29.7)	16.5 (16.0–17.1)	34.3 (33.3–35.3)	68.6 (66.3–70.9)	20.6 (20.0–21.2)	8.0 (7.5–8.6)	26.6 (25.4–27.9)	57.6 (54.6–60.6)	36.5 (35.7–37.4)	23.5 (22.6–24.4)	41.5 (39.9–43.1)	78.4 (74.9–81.9)
Czech Republic	26.0 (25.5–26.5)	15.0 (14.5–15.5)	33.4 (32.4–34.4)	52.9 (50.9–55.0)	20.3 (19.7–20.9)	12.3 (11.6–13.0)	22.9 (21.7–24.1)	47.1 (44.5–49.9)	30.4 (29.6–31.2)	16.9 (16.1–17.7)	41.2 (39.6–42.8)	58.8 (55.9–61.9)
Poland	32.0 (31.5–32.6)	20.0 (19.4–20.7)	42.2 (41.1–43.4)	55.8 (53.7–57.9)	26.0 (25.3–26.8)	18.2 (17.4–19.1)	31.4 (30.0–32.8)	45.0 (42.4–47.7)	36.7 (35.8–37.5)	21.5 (20.6–22.4)	51.2 (49.5–53.0)	62.5 (59.4–65.7)
Luxembourg	19.8 (19.4–20.2)	10.6 (10.1–11.0)	26.9 (26.0–27.8)	40.0 (38.3–41.8)	15.2 (14.6–15.7)	8.3 (7.8–8.9)	19.8 (18.7–20.9)	31.8 (29.6–34.1)	24.7 (24.0–25.4)	12.9 (12.3–13.6)	34.7 (33.3–36.1)	47.8 (45.2–50.6)
Portugal	29.9 (29.3–30.4)	21.0 (20.4–21.6)	34.0 (33.0–35.0)	56.4 (54.3–58.5)	23.2 (22.6–23.9)	16.1 (15.4–16.9)	28.3 (27.0–29.6)	40.0 (37.6–42.6)	35.5 (34.6–36.3)	25.0 (24.1–26.0)	40.4 (38.9–42.0)	66.7 (63.5–69.9)
Slovenia	23.7 (23.2–24.2)	14.1 (13.6–14.7)	27.9 (27.0–28.9)	52.8 (50.8–54.9)	20.5 (19.8–21.1)	11.7 (11.0–12.4)	25.8 (24.6–27.1)	43.5 (40.9–46.1)	25.9 (25.2–26.7)	16.1 (15.4–16.9)	29.4 (28.1–30.7)	58.2 (55.3–61.3)
Estonia	29.0 (28.4–29.5)	19.9 (19.3–20.6)	34.0 (33.0–35.0)	53.6 (51.6–55.7)	24.0 (23.3–24.7)	16.3 (15.5–17.1)	26.9 (25.7–28.2)	48.8 (46.1–51.6)	31.6 (30.8–32.4)	22.1 (21.2–23.0)	37.4 (35.9–38.9)	56.4 (53.5–59.4)
Croatia	21.2 (20.8–21.7)	10.4 (10.0–10.9)	27.5 (26.7–28.5)	50.0 (48.1–52.0)	14.5 (13.9–15.0)	8.8 (8.2–9.4)	14.4 (13.5–15.4)	38.5 (36.1–41.0)	25.8 (25.1–26.6)	11.8 (11.2–12.5)	37.0 (35.5–38.5)	55.6 (52.7–58.6)
TOTAL	23.8 (23.3–24.3)	14.2 (13.7–14.7)	28.7 (27.8–29.7)	51.5 (49.6–53.6)	18.7 (18.1–19.3)	10.9 (10.3–11.6)	21.7 (20.6–22.8)	43.5 (41.0–46.2)	28.0 (27.3–28.8)	16.9 (16.1–17.7)	34.6 (33.2–36.0)	57.6 (54.7–60.7)

**Table 2:** Prevalence of difficulty performing each iADL (%) by country. iADL (Instrumental activity of daily living).

Country	Prevalence of Difficulty Performing Each iADL (%)							
	Doing Work Around the House or Garden	Leaving the House Independently/Accessing Transportation	Shopping for Groceries	Doing Personal Laundry	Managing Money	Preparing A Hot Meal	Taking Medications	Telephone Calls
Austria	17.5	5.7	8.5	6.3	5.2	5.4	1.9	0.9
Germany	14.1	6.9	6.7	5.1	2.5	3.3	1.6	1.3
Sweden	10.8	4.9	3.7	3.5	2.3	1.9	0.8	0.8
Spain	11.4	8.4	5.3	4.3	3.7	3.8	1.7	1.4
Italy	7.6	7.8	5.1	3.7	2.3	1.9	1.1	0.9
France	17.9	8.3	8.9	3.7	4.1	2.2	1.3	2.2
Denmark	13.4	6.1	6.6	4.0	4.2	4.3	1.8	1.3
Greece	13.8	9.5	6.0	4.5	3.4	2.2	1.1	0.4
Switzerland	9.0	4.3	3.9	4.1	2.2	2.0	1.0	1.1
Belgium	20.8	10.4	7.8	4.4	5.7	4.6	1.3	1.7
Israel	21.1	14.0	15.7	10.5	10.4	10.2	2.7	1.5
Czech Republic	16.8	6.6	7.2	5.0	1.7	3.5	1.3	1.2
Poland	22.9	11.2	13.7	8.3	6.0	4.5	1.5	4.2
Luxembourg	12.9	6.4	5.6	3.6	2.4	3.1	2.0	1.1
Portugal	14.3	10.1	8.9	9.0	5.4	5.1	2.2	2.4
Slovenia	12.3	9.1	7.2	4.5	6.6	3.4	1.8	1.6
Estonia	17.0	11.6	11.3	7.1	8.7	5.2	1.3	2.2
Croatia	12.9	8.2	7.5	4.4	7.5	3.7	2.0	1.0
TOTAL	14.6	8.2	7.3	5.0	4.4	3.7	1.5	1.4

**Table 3:** Unadjusted and Adjusted Model. Association of all explanatory variables with one or more iADLs. iADLs (Instrumental activities of daily living); BMI (Body Mass Index).

Explanatory Variable	*N*	*N* (%) Persons with One or More iADL	Unadjusted Model			Adjusted Model		
	27,491	6275 (22.8)	OR	CI 95	*p*	OR	CI 95	*p*
AGE								
≥85 YEARS	2532	1305 (51.5)	1	–	–	1	–	–
75–84 YEARS	9841	2828 (28.7)	0.368	0.336–0.403	<0.001	0.456	0.409–0.508	<0.001
65–74 YEARS	15,118	2142 (14.2)	0.149	0.136–0.164	<0.001	0.256	0.229–0.286	<0.001
GENDER								
FEMALE	15,060	4082 (27.1)	1	–	–	1	–	–
MALE	12,431	2193 (17.6)	0.586	0.553–0.622	<0.001	0.680	0.633–0.730	<0.001
FRAILTY STATUS								
FRAIL	3312	2263 (68.3)	1	–	–	1	–	–
PRE-FRAIL	13,133	3175 (24.2)	0.140	0.128–0.152	<0.001	0.423	0.381–0.470	<0.001
ROBUST	11,046	837 (7.6)	0.035	0.031–0.039	<0.001	0.237	0.206–0.273	<0.001
NETWORK SATISFACTION								
NETWORK SATISFACTION	27,491	6275 (22.8)	0.955	0.936–0.974	<0.001	–	–	–
DEPRESSION								
EURO-D	27,491	6275 (22.8)	1.384	1.366–1.402	<0.001	1.082	1.063–1.101	<0.001
POLYTHERAPY								
NO	18,874	2909 (15.4)	1	–	–	1	–	–
YES	8617	3366 (39.1)	3.585	3.378–3.806	<0.001	1.608	1.493–1.731	<0.001
PHYSICAL INACTIVITY								
NEVER VIGOROUS NOR MODERATE PHYSICAL ACTIVITY	3588	2195 (61.2)	1	–	–	1	–	–
OTHER	23,903	4080 (17.1)	0.120	0.111–0.130	<0.001	0.404	0.365–0.446	<0.001
NUMBER OF CHRONIC DISEASES								
≥2	18,114	5134 (28.3)	1	–	–	1	–	–
<2	9377	1141 (12.2)	0.359	0.335–0.386	<0.001	0.676	0.621–0.736	<0.001
EDUCATION								
NUMBER OF YEARS	27,491	6275 (22.8)	0.915	0.908–0.922	<0.001	0.976	0.967–0.985	<0.001
SELF-PERCEIVED HEALTH								
POOR	3306	1936 (58.6)	1	–	–	1	–	–
FAIR	9999	2784 (27.8)	0.251	0.231–0.273	<0.001	0.507	0.459–0.560	<0.001
GOOD	9949	1290 (13.0)	0.086	0.078–0.095	<0.001	0.304	0.271–0.342	<0.001
VERY GOOD	3310	220 (6.6)	0.039	0.033–0.046	<0.001	0.210	0.176–0.252	<0.001
EXCELLENT	927	45 (4.9)	0.028	0.021–0.039	<0.001	0.176	0.127–0.246	<0.001
BMI								
OBESE	6696	1875 (28.0)	1	–	–	1	–	–
OVERWEIGHT	11,971	2406 (20.1)	0.660	0.616–0.708	<0.001	0.863	0.793–0.940	0.063
NORMAL	8538	1877 (22.0)	0.760	0.705–0.819	<0.001	0.889	0.810–0.976	0.013
UNDERWEIGHT	286	117 (41.0)	1.900	1.488–2.426	<0.001	1.335	0.985–1.809	0.001
SHORTAGE OF MONEY								
NEVER	8768	1839 (21.0)	1	–	–	1	–	–
RARELY	5930	1142 (19.3)	0.898	0.826–0.976	0.012	1.000	0.904–1.105	0.993
SOMETIMES	7163	1581 (22.1)	1.037	0.958–1.122	0.370	1.101	1.000–1.105	0.050
OFTEN	5630	1713 (30.4)	1.550	1.424–1.686	<0.001	1.153	1.039–1.281	0.008
LIFE HAS MEANING								
NEVER	643	298 (46.3)	1	–	–	–	–	–
RARELY	2271	967 (42.6)	0.870	0.729–1.039	0.125	–	–	–
SOMETIMES	7471	2148 (28.8)	0.480	0.407–0.566	<0.001	–	–	–
OFTEN	17,106	2862 (16.7)	0.238	0.203–0.280	<0.001	–	–	–
